# ISLES 2016 and 2017-Benchmarking Ischemic Stroke Lesion Outcome Prediction Based on Multispectral MRI

**DOI:** 10.3389/fneur.2018.00679

**Published:** 2018-09-13

**Authors:** Stefan Winzeck, Arsany Hakim, Richard McKinley, José A. A. D. S. R. Pinto, Victor Alves, Carlos Silva, Maxim Pisov, Egor Krivov, Mikhail Belyaev, Miguel Monteiro, Arlindo Oliveira, Youngwon Choi, Myunghee Cho Paik, Yongchan Kwon, Hanbyul Lee, Beom Joon Kim, Joong-Ho Won, Mobarakol Islam, Hongliang Ren, David Robben, Paul Suetens, Enhao Gong, Yilin Niu, Junshen Xu, John M. Pauly, Christian Lucas, Mattias P. Heinrich, Luis C. Rivera, Laura S. Castillo, Laura A. Daza, Andrew L. Beers, Pablo Arbelaezs, Oskar Maier, Ken Chang, James M. Brown, Jayashree Kalpathy-Cramer, Greg Zaharchuk, Roland Wiest, Mauricio Reyes

**Affiliations:** ^1^University Division of Anaesthesia, Department of Medicine, University of Cambridge, Cambridge, United Kingdom; ^2^Support Center of Advanced Neuroimaging (SCAN), Institute of Diagnostic and Interventional Neuroradiology, University of Bern, Inselspital, Bern University Hospital, Bern, Switzerland; ^3^CMEMS-UMinho Research Unit, University of Minho, Braga, Portugal; ^4^Moscow Institute of Physics and Technology, Dolgoprudny, Russia; ^5^Institute for Information Transmission Problems (RAS), Moscow, Russia; ^6^Instituto de Engenharia de Sostemas e Computadores Investigacã e Desenvolvimento, Lisbon, Portugal; ^7^Department of Statistics, Seoul National University, Seoul, South Korea; ^8^Department of Neurology and Cerebrovascular Center, Seoul National University Bundang Hospital, Seongnam, South Korea; ^9^Department of Biomedical Engineering, National University of Singapore, Singapore, Singapore; ^10^ESAT-PSI, KU Leuven, Leuven, Belgium; ^11^Electrical Engineering and Radiology, Stanford University, Stanford, CA, United States; ^12^Computer Science, Tsinghua University, Beijing, China; ^13^Institute of Medical Informatics, Universität zu Lübeck, Lübeck, Germany; ^14^Biomedical Engineering, University of Los Andes, Bogotá, Colombia; ^15^Athinoula A. Martinos Center for Biomedical Imaging, Harvard, MA, United States; ^16^Department of Radiology, Stanford University, Stanford, CA, United States; ^17^Medical Image Analysis, Institute for Surgical Technology and Biomechanics, University of Bern, Bern, Switzerland

**Keywords:** stroke, stroke outcome, machine learning, deep learning, benchmarking, datasets, MRI, prediction models

## Abstract

Performance of models highly depend not only on the used algorithm but also the data set it was applied to. This makes the comparison of newly developed tools to previously published approaches difficult. Either researchers need to implement others' algorithms first, to establish an adequate benchmark on their data, or a direct comparison of new and old techniques is infeasible. The Ischemic Stroke Lesion Segmentation (ISLES) challenge, which has ran now consecutively for 3 years, aims to address this problem of comparability. ISLES 2016 and 2017 focused on lesion outcome prediction after ischemic stroke: By providing a uniformly pre-processed data set, researchers from all over the world could apply their algorithm directly. A total of nine teams participated in ISLES 2015, and 15 teams participated in ISLES 2016. Their performance was evaluated in a fair and transparent way to identify the state-of-the-art among all submissions. Top ranked teams almost always employed deep learning tools, which were predominately convolutional neural networks (CNNs). Despite the great efforts, lesion outcome prediction persists challenging. The annotated data set remains publicly available and new approaches can be compared directly via the online evaluation system, serving as a continuing benchmark (www.isles-challenge.org).

## 1. Introduction

Defining the location and extent of a stroke lesion is an essential step toward acute stroke assessment. Of special interest is the development of a lesion over time, as this could provide valuable information about tissue outcome after stroke onset. Modern magnetic resonance imaging (MRI) techniques, including diffusion and perfusion imaging, have shown great value to distinguish between acutely infarcted tissue (known as “core”) and hypo-perfused tissue (known as “penumbra”). However, available automated methods used to characterize core and penumbra regions from MRI information lack accuracy and cannot correctly capture the complexity of the image information. Hence, there is a great need for advanced data analysis techniques that identify these regions and predict tissue outcome in a more reproducible and accurate way. Eventually, such tools will be available to support clinicians in their decision-making process (e.g., deciding for or against thrombolytic therapy). In recent years machine learning methods for medical image computing have shown unprecedented levels of progress. The area of supervised machine learning (i.e., where computer models are trained based on existing pre-annotated datasets) and particular deep learning, has gained much popularity and has shown great potential for medical applications where image quantification and interpretation is important for the decision making process (1). Along with this, the benchmarking of machine learning techniques for medical image computing has become a central area of interest at the annual Medical Image Computing and Computer Assisted Intervention (MICCAI) conference, where algorithms are tested and evaluated using curated datasets and common evaluation metrics. The ISLES challenge was created as an effort to raise the interest of the medical image computing community to make progress on the challenging aspects of stroke lesion characterisation from MRI data. The work of Maier and colleagues summarizes the lessons learned from the ISLES 2015 edition (2), where image analysis at the subacute and acute stages provided insights as to how accurate machine learning approaches could characterize core and penumbra regions. In the following years the discussions happening among interdisciplinary teams at the ISLES challenge, allowed the community to advance toward the challenge of stroke lesion prediction from MRI data. This is of great interest in a clinical routine, as the responsible physician needs to decide quickly, whether the particular stroke patient could benefit from an interventional treatment (i.e., thrombectomy or thrombolysis). This decision is often draw on basis of lesion appearance, the time passed since stroke onset and the clinicians personal experience. Objective methods that reliably predict lesions and clinical outcome only from the acute scans would be a powerful tool to support and accelerate decision making during the critical phase.

### 1.1. Current methods

From the literature review presented by Maier et al. (2), summarizing the state of the art until 2016, the recent machine learning methods for stroke lesion segmentation and outcome prediction clearly show the transition from classic machine learning tools [e.g., (3, 4)] to approaches based on deep learning (5–10). Generally, the accuracy of those methods is tightly connected to the data set they have been applied to and prevent a direct comparison. For this reason, the development of a publicly available benchmarking, such as ISLES is crucial to facilitate the analysis of current machine learning technologies and leverage research lines to improve them. The ISLES challenge held in 2016 and 2017 have hosted a total of 24 teams participating in the lesion segmentation and outcome prediction sub-tasks. In this article, we present the main results and findings in benchmarking machine learning approaches presented at ISLES 2016 and 2017. The ISLES challenges feature 75 cases from two different centers, including perfusion and diffusion imaging (Raw Perfusion, CBF, CBV, TTP, Tmax, ADC, MTT) as well as clinical information (time-since-stroke, time-to-treatment, TICI and mRS scores). Through reference annotations produced by two clinical experts, and a set of quantitative metrics and qualitative expert evaluations, we analyse and describe common strategies and approaches leading to best algorithmic performance. We present the progress of these algorithms, and current challenges that these techniques need to overcome in order to integrate them into the time-critical clinical workflow of stroke patients.

### 1.2. Motivation for ISLES and challenge setup

Automated methods for lesion segmentation and prediction are part of an active research field. Since results are highly dependent on the size and quality of the used data, comparison of independent validation methods is challenging. In order to compare different automated methods, researchers typically have to reimplement algorithms presented in previous publications, which is known to be a difficult task due to the complexity of the algorithms, and lack of detailed description of their implementation. Although the community is changing and provides more frequently open source code is more frequently provided, benchmarking remains time consuming. For these reasons, computational challenges aim to provide a platform allowing a fair and on going validation of various methods tackling a predefined problem. The ISLES challenge follows this direction by providing a stroke imaging database and benchmarking platform that facilitates the comparisons of new algorithms for lesion segmentation and prediction. ISLES was launched for the first time in 2015 and was successfully continued in the subsequent 2 years. Researchers interested in this challenge could register online and download the imaging data via the SICAS Medical Image Repository (SMIR) platform (11). The training data was provided in a preprocessed format that allowed teams to apply their algorithms directly without need of pre-processing. Furthermore, this ensured that performance differences are mainly driven by the prediction models, rather than different preprocessing steps. Eventually, methods could be directly compared and ranked on a leaderboard to discover the most successful approach.

#### 1.2.1. ISLES 2016

While the focus for ISLES 2015 lied on ischemic stroke lesion segmentation (2), ISLES 2016 aimed for the outcome prediction of lesions. Therefore, multispectral MRI data from acute phase of 35 stroke patients were provided together with lesion maps annotated on 3–9 month follow-up scans. After a period of several weeks, participating teams (See Table 1) were asked to apply their algorithm to 19 unseen test data. The lesion labels for the test data were generated by two raters independently, and merged via the STAPLE algorithms (12) to generate a fused ground-truth dataset. On basis of the performance on this test data set, methods were ranked to define a winner of the challenge. As a second task, teams were asked to predict the clinical mRS score, which denotes the degree of disability. Upon analysis of the results, we acknowledge that the latter task required more data for a reliable statistical analysis, which is why they are not presented in this paper. However, the reader is referred to the official website for ISLES 2016^1^ for more details.

**Table 1:** Participants of ISLES 2016 (more details and main features of each method see Appendix ISLES16-A1 to ISLES16-A7).

CH-UBE	University of Bern, Switzerland
	Incorporating time to reperfusion into the FASTER (3) model of stroke tissue-at-risk
DE-UZL	Institute of Medical Informatics, Universität zu Lübeck, Germany
	Random forests for stroke lesion and clinical outcome prediction
HK-CUH	Deptartment of Computer Science and Engineering, The Chinese University of Hong Kong
	Residual Volumetric Network for Ischemic Stroke Lesion Segmentation
KR-SUC^*^	Department of Statistics, Seoul National University, Korea
KR-SUK^*^	Deep Convolutional Neural Network Approach for Brain Lesion Segmentation
KR-SUL^*^	
PK-PNS	Pakistan Institute of Nuclear Science and Technology, Islamabad, Pakistan
	Segmentation of Ischemic Stroke Lesion using Random Forests in Multi-modal MRI Images
UK-CVI	CVIP, Comp. at School of Science and Eng., University of Dundee, UK
	Combination of CNN and Hand-crafted feature for Ischemic Stroke Lesion Segmentation
US-SFT	University of Southern California, Fractal Analytics, TopicIQ
	A Deep-Learning Based Approach for Ischemic Stroke Lesion Outcome Prediction

* *These methods are variants of a single method*.

#### 1.2.2. ISLES 2017

Similarly to ISLES 2016, in 2017 participants were asked to predict lesion outcome on MRI data. The data set of ISLES 2016 was expanded to a total of 43 patients for the training phase, and 32 cases for methods evaluation (see Table 3). For the additional 13 test cases, added in 2017, only one groundtruth was generated (in contrast to the other 19 cases from ISLES 2016, for which two annotations per cases exist). For ISLES 2017, participants were asked to submit an abstract, describing their approach, until August 2017. Mid August the test data was distributed and teams had the chance to apply their models and submit their final prediction 2 weeks later. Participating teams and their submitted abstract titles can be found in Table 2, along with main features of each method (detailed description of methodology in Appendix).

**Table 2:** Participants of ISLES 2017 (more details and main characteristic of each method see Appendix ISLES17-A1 to ISLES17-A14).

AAMC	Athinoula A. Martinos Center, USA
	Ensembling 3D U-Nets For Ischemic Stroke Lesion Segmentation
HKU-1	Hong Kong University of Science and Technology, China
	Deep Adversarial Networks for Stroke Lesion Segmentation
HKU-2	Hong Kong University of Science and Technology, China
	Stochastic Dense Network for Brain Lesion Segmentation
INESC	INESC-ID, Portugal
	Fully Convolutional Neural Network for 3D Stroke Lesion Segmentation
KU	Korea University, Korea
	Gated Two-Stage Convolutional Neural Networks for Ischemic Stroke Lesion Segmentation
KUL	KU Leuven, Belgium
	Dual-scale Fully Convolutional Neural Network for Final Infarct Prediction
MIPT	Moscow Institute of Physics and Technology, Russia
	Neural Networks Stroke Lesion Segmentation
NEU	NEUROPHET Inc. Seoul, South Korea
	Combination of U-Net and Densely Connected Convolutional Networks
NUS	National University of Singapore, Singapore
	Fully Convolutional Network with Hypercolumn Features for Brain Lesion Segmentation
SNU-1^*^	Seoul National University, Korea
SNU-2^*^	Schemic Stroke Lesion Segmentation with Convolutional Neural Networks for Small Data
SU	Stanford University, USA
	Multi-scale Patch-wise 3D CNN for Ischemic Stroke Lesion Segmentation
UA	Universidad de los Andes, Colombia
	Volumetric Multimodality Neural Network For Ischemic Stroke Segmentation
UL	University of Luebeck, Germany
	2D Multi-Scale Res-Net for Stroke Segmentation
UM	Universito of Minho, Portugal
	Combining Clinical Information for Stroke Lesion Outcome Prediction using Deep Learning

* *These methods are variants of a single method*.

The access to the ISLES data remains open so that future research efforts can easily be compared against the existing benchmark.

### 1.3. Data and methods

#### 1.3.1. Data acquisition and pre-processing

Subjects used for the database, were patients treated for acute ischemic stroke at the University Hospital of Bern or at the UMC Freiburg between 2005 and 2015. Diagnosis of ischemic stroke was performed by identification of lesions on DWI and PWI MR imaging. Digital subtraction angiography was employed to document proximal occlusion of the middle cerebral artery (M1 or M2 segment).

Patient inclusion criteria considered: (I) Identification of ischemic stroke lesions on DWI and PWI imaging, (II) proximal occlusion of the middle cerebral artery (M1 or M2 segment) documented on digital subtraction angiography, (III) attempt for endovascular therapy was undertaken, either by intra-arterial thrombolysis (before 2010) or by mechanical thrombectomy (since 2010), (IV) no motion artifacts during pretreatment MR imaging, and (V) patients had a minimum age of 18 years at the time of stroke. Patients were excluded if they had undergone a purely diagnostic angiography and if stenosis or occlusion of the carotid artery were found.

MR imaging was performed on a 1.5T (Siemens Magnetom Avanto), and on a 3T MRI system (Siemens Magnetom Trio). The stroke protocol encompassed whole brain DWI, (24 slices, thickness 5mm, repetition time 3,200ms, echo time 87ms, number of averages 2, matrix 256^*^256) yielding isotropic b0 and b1000 as well as apparent diffusion coefficient maps (ADC) that were calculated automatically. Additionally, a T2 image was acquired for each case, which was not released to participants but used later for the generation of the groundtruth lesion outcome delineations (section 1.3.2) For PWI, the standard dynamic-susceptibility contrast enhanced perfusion MRI (gradient-echo echo-planar imaging sequence, repetition time 1,410ms, echo time 30ms, field of view 230^*^230mm, voxel size: 1.8^*^1.8^*^5.0mm, slice thickness 5.0mm, 19 slices, 80 acquisitions) was acquired. PWI images were acquired during first pass of a standard bolus of 0.1mmol/kg gadobutrol (Gadovist, Bayer Schering Pharma, Berlin, Germany). Contrast medium was injected at a rate of 5ml/s followed by a 20ml bolus of saline at a rate of 5ml/s. Perfusion maps were obtained by block-circular singular value decomposition using the Olea-Sphere software v2.3(Olea Medical, La Ciotat). Raw PWI images were also released to participants in the form of a single 4D NifTI image, to allow teams interested in using a different parametric map reconstruction method. All PWI maps (rBF, rBV, TTP, Tmax, MTT) were rigidly registered to the ADC image and automatically skull-stripped (2) to extract the brain area only. We remark, this alignment step was performed to standardize the pre-processing step, hence, to factor out this pre-processing step from the evaluation of results. The cohort curated in 2016 was then extended into a larger dataset for the challenge in 2017. Table 3 summarizes the ISLES 2016 and 2017 dataset.

**Table 3:** Details of the ISLES 2016 & 2017 Data.

	2016	2017
Number of cases	35 training and 19 testing	43 training and 32 testing
Number of expert segmentations for training and testing sets	1 (training), 2 (testing)	1 (training), 1 (testing)
MRI sequences	ADC, rBF, rBV, MTT, TMAX, TTP, Raw PWI	ADC, rBF, rBV, MTT, TMAX, TTP, Raw PWI

#### 1.3.2. Groundtruth lesion outcome segmentation

The lesion outcome status was manually segmented by a board-certified neuroradiologist using 3D Slicer v4.5.0-1, and based on the 90-day follow-up T2 image. Regions of maximal extent of the final infarction, including haemorrhagic transformation but excluding hyper-intense areas on the acute T2 image (i.e., infarctions due to previous CVI), were delineated on every transversal slice. The 90-day follow-up lesion was chosen to be delineated, since it yields a more reliable final lesion volume than the apparent lesion volume that is observable on subacute images. Groundtruth images were converted into the NIfTI format for distribution to participants. For the 19 test cases of ISLES 2016, two lesion annotations were generated by individual raters, and subsequently merged via STAPLE algorithm (12).

#### 1.3.3. Lesion characteristics

We performed a correlation analysis to assess a possible connection between clinical variables and the performance of the automated methods. The evaluation was conducted for ISLES 2017 submitted methods. Table 4 summarizes the collected information.

**Table 4:** Summary of lesion characteristics for ISLES 2017 Data.

Lesion count	mean [min, max] = 2.46[1, 14]
Lesion volume	mean [min, max] = 37.83*ml*[1.6*ml*, 160.4*ml*]
Lesion localisation in Lobes	for all 32 cases lesions were located in more than one lobe
Lesion localisation	*n*_*subcortical*_=3, *n*_*cortical*_=29
Involved territory	*n*_*MCA*_=29, *n*_*MCA*+*PCA*_ = 1, *n*_*multiple*_=1
Midline shift	not present for any of the 32 cases
Laterality	*n*_*left*_=16, *n*_*right*_=16
White matter lesions^*^	*n*_0_=9, *n*_1_=10, *n*_2_=8, *n*_3_=5

*n, number of cases with given feature; MCA, middle cerebral artery, PCA, posterior cerebral artery*.

* *Fazekas Classification: 0, absent; 1, punctuate; 2, beginning confluent areas; 3, large confluence*.

#### 1.3.4. Evaluation metrics

As quantitative evaluation metrics of the presented methods, we calculated the Dice score as a measure of overlap between manually outlined and automatically predicted lesions. To further shed light on the algorithm's effect we computed precision and sensitivity scores. With TP, true positives; FP, false positive and FN, false negative; the metrics were defined as followed:

(1) $$\begin{array}{r} Dice=\frac{2TP}{2TP+FP+FN} \end{array}$$

(2) $$\begin{array}{r} Precision=\frac{TP}{TP+FP} \end{array}$$

(3) $$\begin{array}{r} Sensitivity=\frac{TP}{TP+FN} \end{array}$$

Alongside these, we measured the maximum surface distance between automatically defined volume and the manually delineated groundtruth volumes by means of the Hausdorff distance (HD). Denoting *A*_*S*_ and *B*_*S*_ as the surface voxels of groundtruth and segmentation volume, respectively, we calculated:

(4) $$\begin{array}{r} HD\left(A_{S},B_{S}\right)=\max\left\{\underset{a\epsilon A_{S}}{\max}\;\underset{b\epsilon B_{S}}{\min}\;d\left(a,b\right),\;\underset{b\epsilon B_{S}}{\max}\;\underset{a\epsilon A_{S}}{\min}\;d\left(b,a\right)\right\} \end{array}$$

As distance measure *d*(·, ·) we used the Euclidean distance.

Additionally, the average symmetric surface distance (ASSD) was computed for ISLES 2016:

(5) $$\begin{array}{r} ASSD\left(A_{S},B_{S}\right)=\frac{ASD\left(A_{S},B_{S}\right)+ASD\left(B_{S},A_{S}\right)}{2} \end{array}$$

with the average surface distance (ASD) defined as:

(6) $$ASD(A_{S},B_{S})=\frac{\sum_{a\epsilon A_{s}}min_{b\epsilon B_{s}}d\left(a,b\right))}{\left|A_{S}\right|}$$

#### 1.3.5. Ranking approach

**Figure 1:**
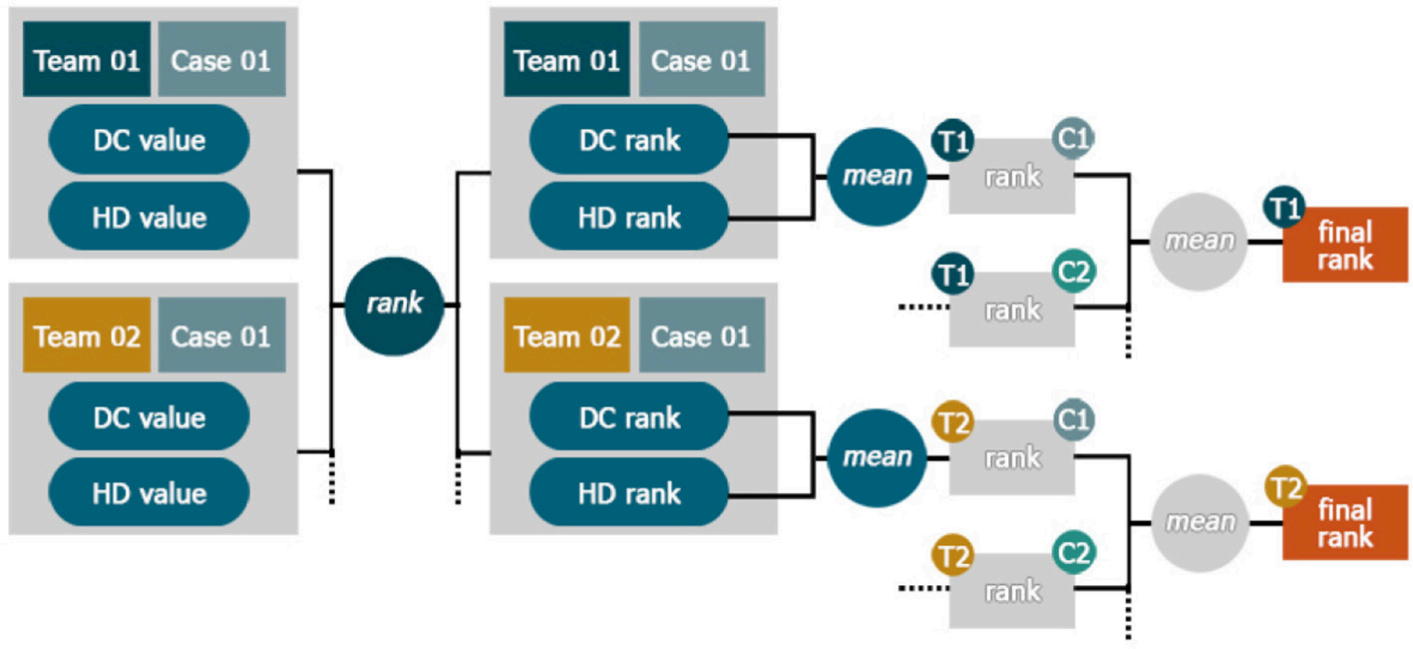
Ranking Scheme. The teams were sorted by their different performance metrics e.g., Dice score (DC) and assigned a rank value per case. Ranks for each team were then separately averaged on a case-wise basis. The final team's rank was then calculated as the mean of all its case-ranks.

In order to rank participant's submission for ISLES 2017, we focused on Dice score, as it combines both precision and sensitivity into one metric, and the HD metric. First, both measurements were computed for each patient data individually. Then, all participants were ranked for each metric separately on a case-wise basis such that a high Dice score and a low HD resulted in a high rank. The mean of both ranks yielded a case specific rank. A participant's total rank is obtained by averaging the ranks over all cases (see Figure 1). Ranks for ISLES 2016 were computed in the same way for both available groundtruths. Furthermore, ASSD was included alongside Dice and HD for ISLES 2016. In case where teams were not submitting all testing results, the Dice scores were completed with 0 and a large (i.e., 1e+5) value was set for HD. All unsuccessful segmentation (Dice = 0), were always ranked last. Segmentations with the exact same metrics received the same rank.

#### 1.3.6. Fusion and thresholding of softmax maps

Fusing the output of several classifiers has been shown to yield better results than the single classifiers. This concept is the foundation for ensemble learners, such as random forest (13), and has also been shown to be beneficial for tumor lesion segmentation (14, 15). In theory, each different model could provide valuable, complementary information to enhance the overall segmentation performance. All submitted methods for ISLES 2017 were deep neuronal networks. These include by design a final classification layer, which is commonly a softmax function that provides voxel-wise output values between [0, 1] (further referred to as softmax maps). This output can be interpreted as a probability of voxel belonging to its given class (in this case healthy or lesion tissue). To leverage potential benefit of several submitted models, we averaged the softmax maps of the top five and top three ranked methods for each individual case, followed by its thresholding at the 0.5 mark. Moreover, the softmax maps were thresholded at various levels and subsequently binarised in order to analyse the robustness of methods. Finally, the Dice score was computed between these binary images and the groundtruth.

#### 1.3.7. Statistical analysis

To assess statistical differences between the submitted methods we applied a Friedman test, a non-parametric, one-way analysis of variances for repeated measurements, and *post-hoc* Dunn test for multiple comparison between teams. For all tests we used GraphPad PRISM Version 5.0.1. The levels of significant differences are marked in plots with asterisks (^*^p < 0.05, ^*^^*^p < 0.01, and ^*^^*^^*^p < 0.001).

## 2. Results

### 2.1. ISLES 2016

#### 2.1.1. Inter-observer variance

The annotated volumes by rater 1 range from median [Q1, Q3] = 16.7 [6.1, 41.6] ml, and for rater 2 from median [Q1, Q3] = 9.0 [2.9, 36.8] ml, revealed the tendency of rater 1 having segmented more tissue as lesion than rater 2. In 18 out of 19 cases, rater 1 outlined larger lesion volumes, which holds true especially for rather small lesions. Comparing the overlap between manually outlined lesions of both raters yielded an average Dice score of 0.58 ± 0.20, with median [Q1, Q3] = 0.62 [0.39, 0.77]. The relative low coherence between the experts' annotations shows shows the difficulty of outlining the follow-up image.

#### 2.1.2. Leaderboard and statistical analysis

Table 5 shows the ranking of the submitted methods. Only four (KR-SUC, CH-UBE, HK-CHU, PK-PNS) out of nine teams managed to get a successful lesion prediction (Dice > 0) for all 19 cases. The ranking reflects mostly the teams' Dice ranks, except for CH-UBE which was ranked on fourth place despite the second lowest average Dice score (not shown in table). This can be explained by the relative good HD (not shown in table) in comparison to the last ranked teams (see Table 5 places 7–9).

**Table 5:** Leaderboard ISLES 2016: The rank specifies the final value to order methods relative to each other by performance.

**Place**	**Team**	**Rank**	**Dice rank**	**HD rank**	**ASSD rank**	**Cases**
1	KR-SUL	3.03	**3.37**	**2.79**	**2.92**	18/19
2	KR-SUC	3.57	3.58	3.71	3.42	18/19
3	KR-SUK	3.82	3.74	4.13	3.61	**19/19**
4	CH-UBE	3.95	4.26	3.76	3.82	**19/19**
5	DE-UZL	4.21	4.21	3.82	4.61	**19/19**
6	UK-CVI	4.08	5.11	4.68	5.45	16/19
7	HK-CHU	5.59	5.08	5.53	6.16	**19/19**
8	PK-PNS	6.48	6.34	7.58	5.55	12/19
9	US-SFT	8.07	8.03	8.03	8.16	11/19

*Dice, HD, and ASSD rank are the average achieved ranks for each participating team per case. The last column gives the number of successfully (Dice > 0) predicted lesions. Best mean values printed in bold*.

**Figure 2:**
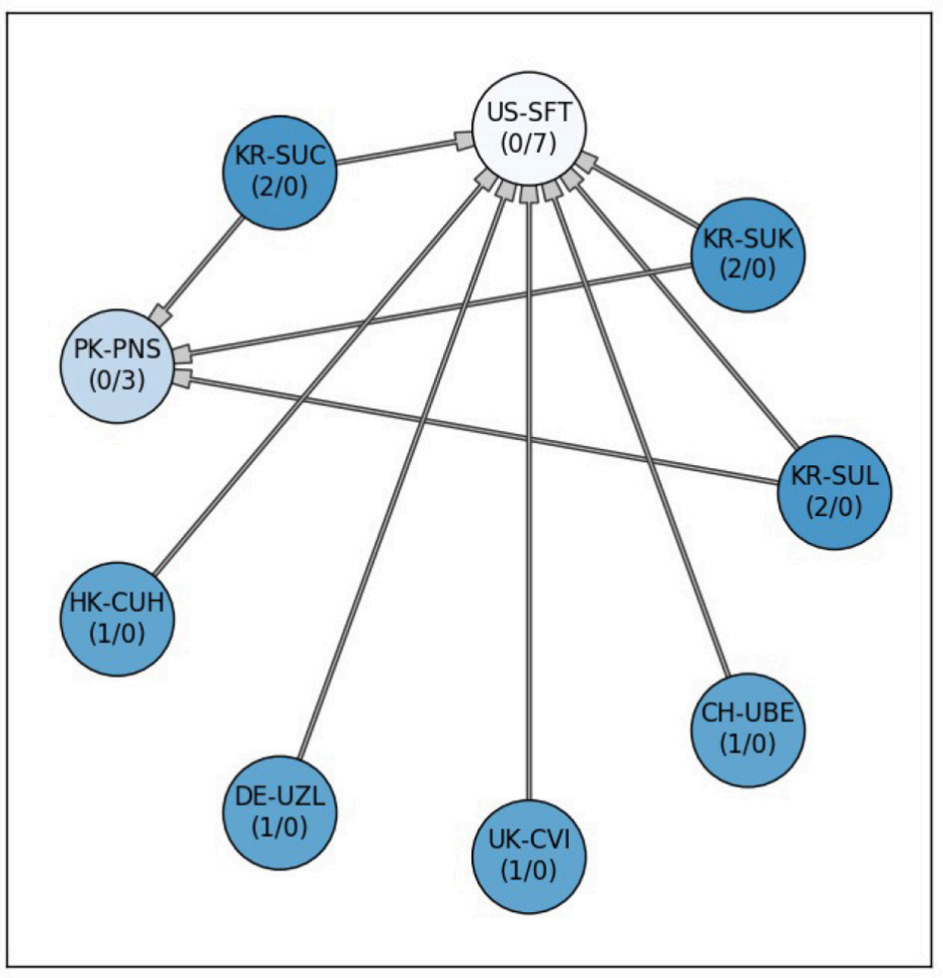
Significant differences between the 9 submitted methods for ISLES 2016. Each node stands for one participating team. A connection between the nodes represents a significant difference between both lesion prediction models. Methods at the tail side of the arrow indicate superiority to the corresponding connected one. The stronger or weaker a model is the more outgoing or incoming connections (#outgoing/#incoming, respectively), are associated with a team's node. Additionally, the node's color saturation indicates the strength of a method (differences in Friedman test rank sum), with better methods appearing more saturated (i.e., darker blue). All methods, except for PK-PNS, are significantly better than US-SFT (*post-hoc* Dunn test *p* < 0.05).

Analysing the Dice scores across all methods showed that almost all methods are superior to that of US-SFT, which was ranked last. Only PK-PNS, which came second to last, was not found statistically different from US-SFT. The winning approaches (KR-SUC, KR-SUK, KR-SUL) achieved also significantly higher Dice scores than PK-PNS. All methods ranked in second cluster of groups(CH-UBE, DE-UZL, HK-CUH, UK-CVI) did not show statistically significant differences to one and another (see Figure 2).

**Figure 3:**
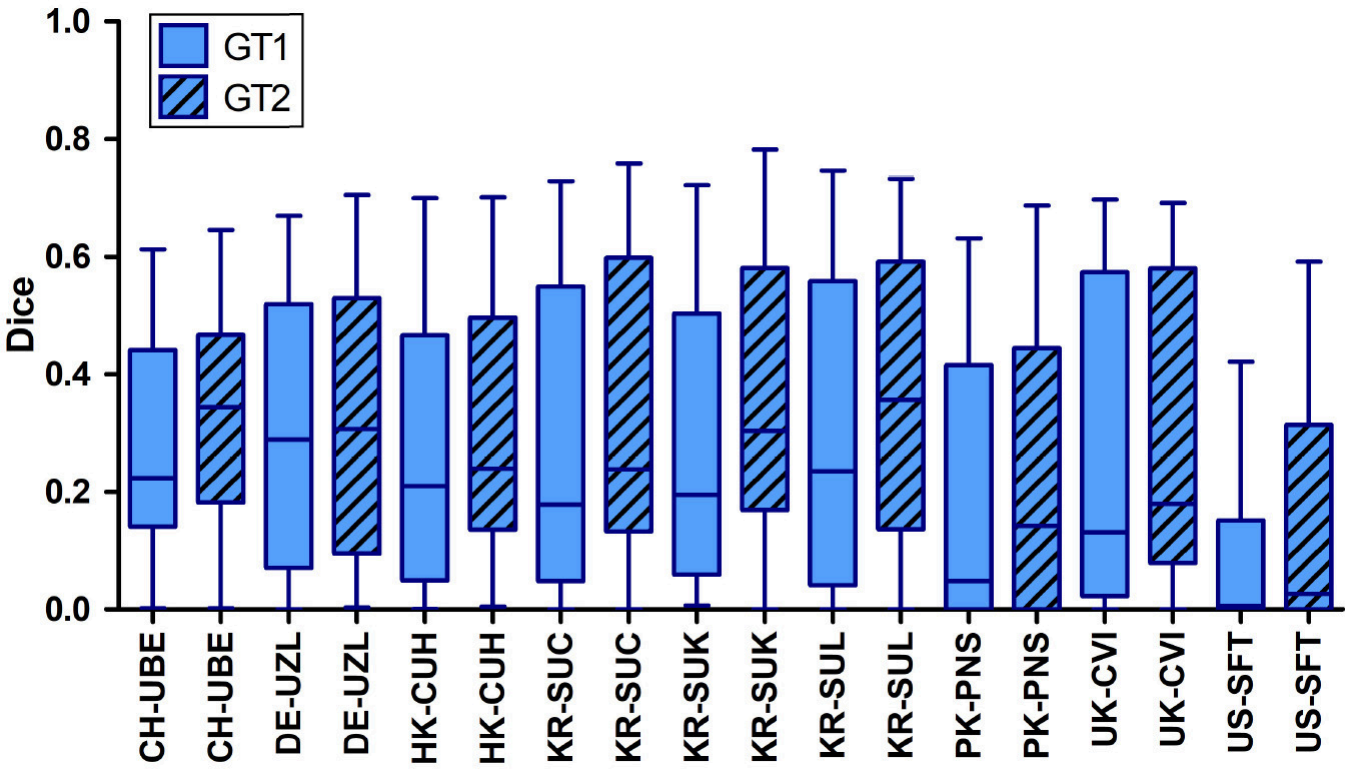
Distribution of Dice scores computed between the automatic lesion predictions and both groundtruths (GT1 and GT2) individually for ISLES 2016. For all teams the Dice scores computed with respect to rater 1 were significantly lower than those calculated with respect to the 2nd groundtruth (GT2).

Comparing the Dice scores directly for both manual annotations individually, revealed a positive bias toward the groundtruth generated by the second rater. For all teams the average Dice for both groundtruths varied around five percentage points (see Figure 3).

### 2.2. ISLES 2017

#### 2.2.1. Leaderboard

Only one (SU) of the 15 teams was able to predict stroke lesions (Dice > 0) consistently for all 32 cases. Examining the average Dice and HD rank for each time separately, revealed that the second ranking team (UL) yielded a lower Dice rank than the following two teams (i.e., HKU-1 and INESC). However, UL achieved the best HD rank, which secured its second place (see Table 6).

**Table 6:** Leaderboard ISLES 2017: While the rank denotes the final value used to sort the teams performances relative to each other.

**Place**	**Team**	**Rank**	**Dice rank**	**HD rank**	**Cases**
1	SNU-2	5.25	**4.53**	5.97	30/32
2	UL	5.42	6.16	**4.69**	29/32
3	HKU-1	5.55	5.09	6.00	29/32
4	INESC	5.92	5.00	6.84	31/32
5	KUL	6.03	6.19	5.88	30/32
6	SNU-1	6.47	6.25	6.69	29/32
7	UM	6.58	6.31	6.84	31/32
8	MIPT	6.72	6.34	7.09	30/32
9	SU	7.20	7.09	7.31	**32/32**
10	KU	8.75	10.09	7.41	28/32
11	AAMC	9.05	8.63	9.47	27/32
12	UA	9.78	9.31	10.25	29/32
13	NUS	9.95	9.50	10.41	29/32
14	NEU	10.44	11.88	9.00	16/32
15	HKU-2	11.80	12.50	11.09	14/32

*Dice and HD rank are the average achieved ranks for each participating team. The cases column denotes the number of successfully (DC > 0) predicted lesions. Best mean values printed in bold*.

#### 2.2.2. Dice, precision and sensitivity

Table 7 summarizes the participating teams' performance, measured by Dice score, precision and sensitivity, highlighting the strengths of different models. Team KUL's model was the most precise while showing lower sensitivity. AAMC's model showed the highest sensitivity while lacking in precision. Although HKU-1 achieved the highest mean Dice score, it was ranked third seemingly due to a lower HD rank (compare Table 6). Even top ranking models reached a low average Dice score of around 0.3, underlining the substantial difficulty of lesion forecasting.

**Table 7:** Average Dice score, precision and sensitivity for individual teams across all 32 cases for ISLES 2017.

**Place**	**Team**	**Dice**	**Precision**	**Sensitivity**
1	SNU-2	0.31 ± 0.23	0.36 ± 0.27	0.45 ± 0.31
2	UL	0.29 ± 0.21	0.34 ± 0.26	0.51 ± 0.33
3	HKU-1	**0.32** ± 0.23	0.34 ± 0.27	0.39 ± 0.28
4	INESC	0.30 ± 0.22	0.34 ± 0.27	0.51 ± 0.31
5	KUL	0.27 ± 0.22	**0.44** ± 0.33	0.39 ± 0.31
6	SNU-1	0.28 ± 0.23	0.36 ± 0.31	0.41 ± 0.31
7	UM	0.29 ± 0.22	0.26 ± 0.24	0.61 ± 0.28
8	MIPT	0.27 ± 0.20	0.31 ± 0.28	0.39 ± 0.29
9	SU	0.26 ± 0.21	0.28 ± 0.25	0.56 ± 0.26
10	KU	0.17 ± 0.16	0.23 ± 0.28	0.36 ± 0.33
11	AAMC	0.23 ± 0.22	0.19 ± 0.20	**0.62** ± 0.37
12	UA	0.19 ± 0.16	0.27 ± 0.25	0.21 ± 0.18
13	NUS	0.19 ± 0.16	0.29 ± 0.26	0.23 ± 0.22
14	NEU	0.11 ± 0.16	0.17 ± 0.25	0.12 ± 0.17
15	HKU-2	0.05 ± 0.10	0.17 ± 0.28	0.05 ± 0.11

*All evaluation measures are given in mean ± standard deviation. Best mean values printed in bold*.

Analysing the Dice score per case disclosed a wide range of quality of lesion outcome prediction. While there are a few cases (28–32) where the average Dice score was above 0.5, the majority of cases turned out to be hard to predict. For 14 cases at least one team achieved a prediction that was overlapping with the groundtruth by 50% (Figure 5). For six cases (1–5, 9) none of the teams reached the overall mean Dice score (0.23).

**Figure 4:**
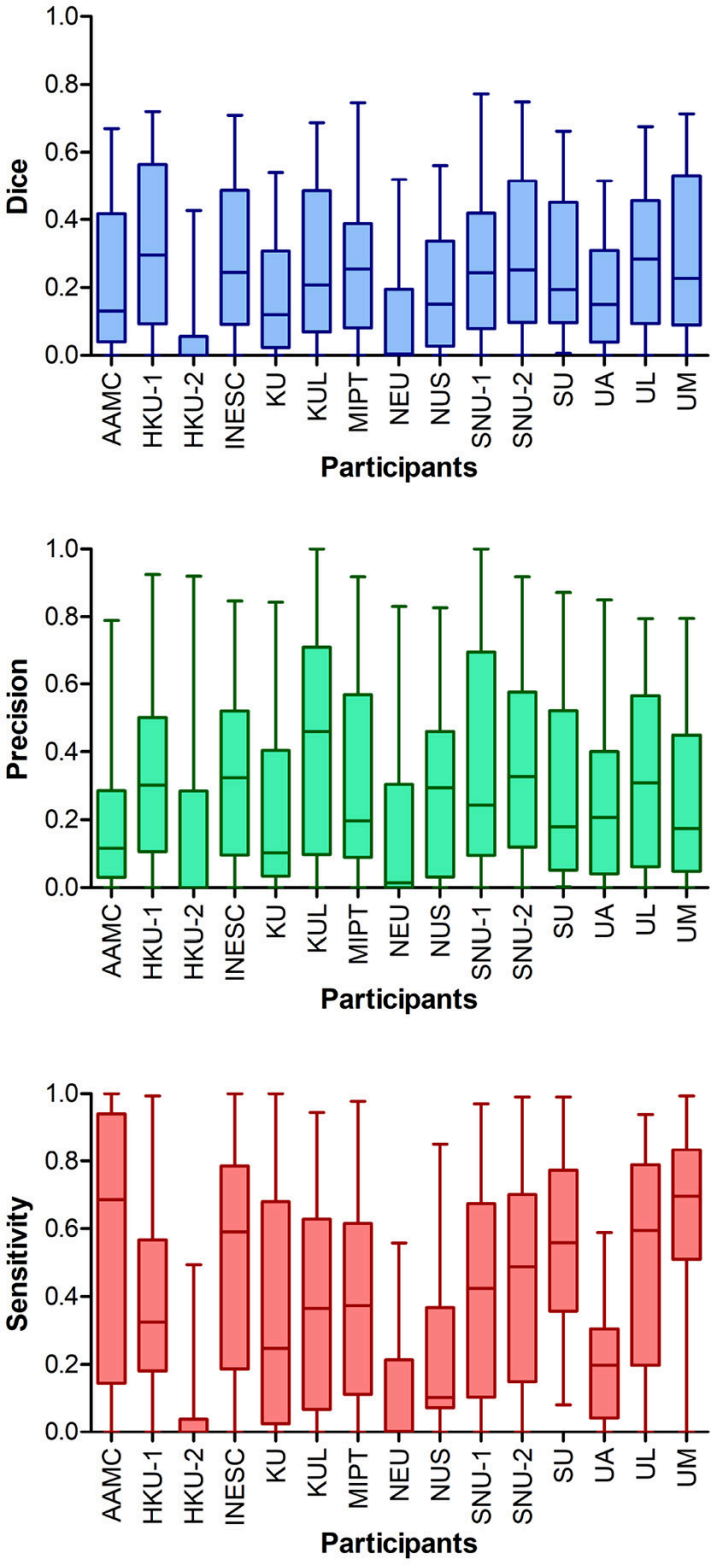
Performance metrics for all teams of ISLES 2017. Higher ranking teams (e.g., 1st place SNU-2) achieved Dice scores > 0.7 for some cases, however, overall Dice scores clustered around 0.2–0.3. The two teams ranked last (NEU and HKU-2) showed much lower Dice scores than all other teams, which was a consequence of the low number of successful submissions. The model of UM seemed to be most sensitive to detect lesions, but lacks in precision.

**Figure 5:**
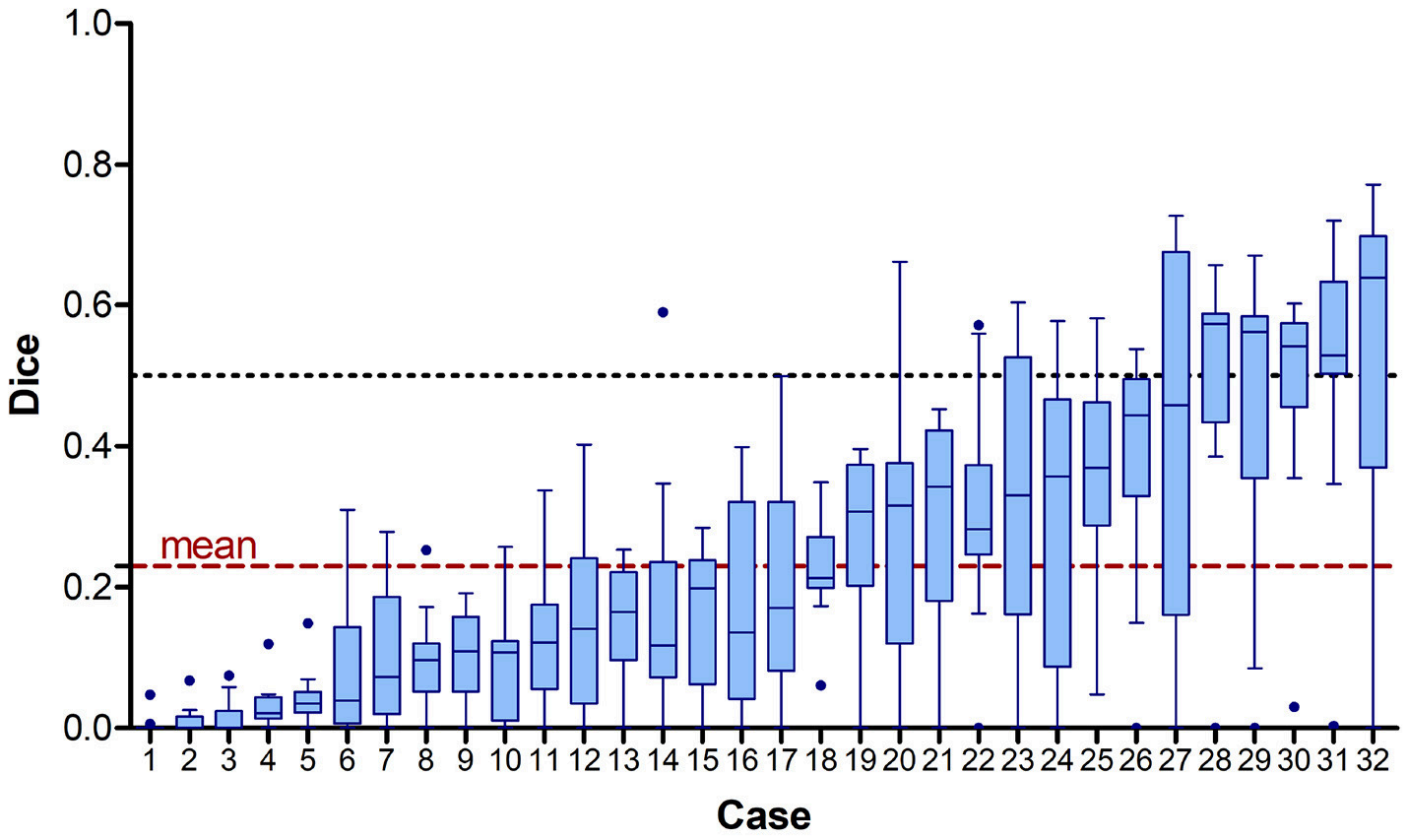
Achieved Dice scores for each case across all 15 participating teams sorted by mean value. The dashed line shows the overall mean Dice score of 0.23 (red) and the 0.5 mark (black). Note that the case numbers were assigned according to ascending mean Dice score.

#### 2.2.3. Statistical comparison of team performances

**Figure 6:**
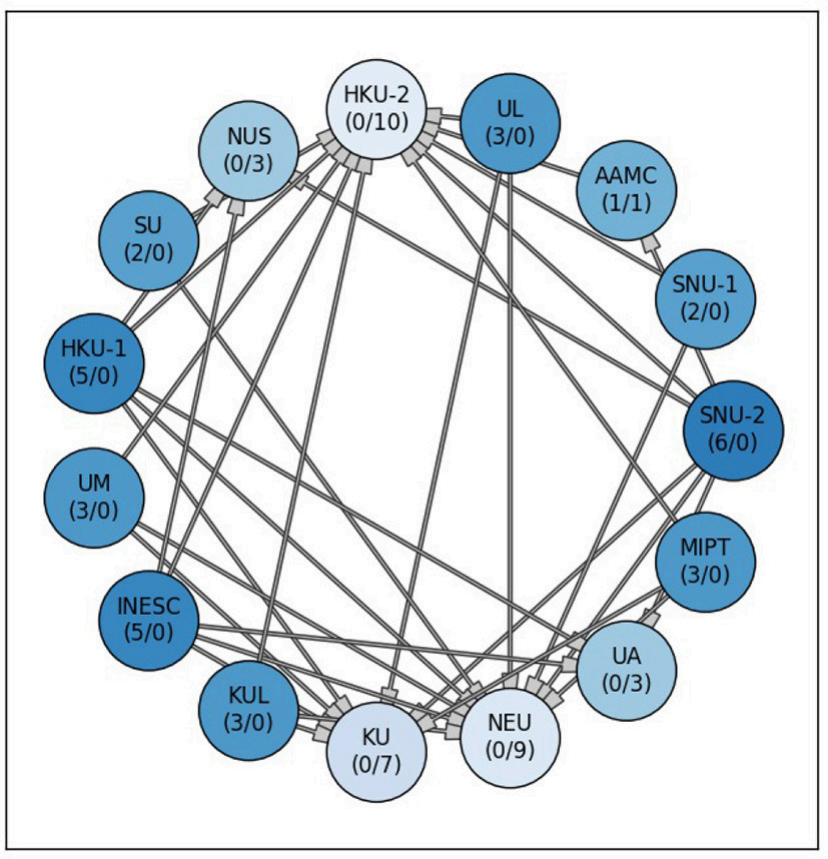
Significant differences between the 15 submitted methods at ISLES 2017. Each node stands for one participating team. A connection between two nodes represents a significant difference between both lesion prediction models, whereas the methods at the tail side was superior. The stronger or weaker a models the more outgoing or incoming connections (#outgoing/#incoming), are associated with a team's node. Additionally, the node color saturation indicates the strength of a method, with better methods appearing more saturated. Differences between methods were assessed via non-parametric ANOVA with repeated measurements (Friedman test) and subsequent, pair-wise comparison with Dunn test (*p* < 0.05).

Figure 6 shows the comparison of the team's Dice scores on the test data set. Each method, represented as node, connects to other methods when a statistical differences in the Dice scores was found. Methods associated to nodes with more outgoing and less incoming connections can be considered stronger than other with less outgoing or more incoming connections. The nodes for stronger models were further grouped and indicated by a more saturated color. This visually highlights the winning team SNU-2 that showed overall higher Dice scores for the prediction lesions than the other six teams, while none of the other methods were significantly better. This is closely followed by HKU-1 and INESC having each five outgoing edges. The two worst methods (NEU, HKU-2) failed to predict the lesions for several subjects completely, resulting in poor performance inferior to most teams (9 and 10 respectively).

#### 2.2.4. Performance of single models vs. ensembles

As mentioned in section 1.3.6 we fused the softmax maps to create an ensemble of the top five (E5 = SNU-1, SNU-2, UL, INESC, KUL) and top three (E3 = SNU-2, UL, INESC) ranking teams^2^ and compared both ensembles to their individual models. All included models had no significantly different Dice score distributions in comparison to each other (see Figure 6).

**Figure 7:**
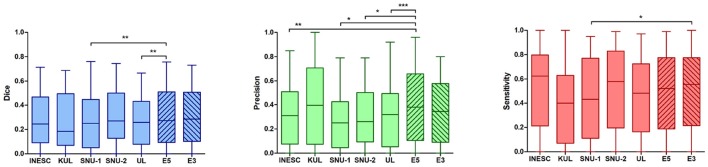
Statistical comparison of lesion prediction performance of single models vs. ensembles. **Left**: An ensemble of five models (E5) could improve the Dice score in comparison to the two weaker models (SNU-1 *p* < 0.01, UL *p* < 0.05). This effect was, however, not observed when building an ensemble with three models (E3). **Middle**: The ensemble E5 significantly gained precision in contrast to most of the single models (SNU-1 *p* < 0.01, SNU-2 *p* < 0.05, UL *p* < 0.001, INESC *p* < 0.01). KUL's precision was higher or similar to that of the ensembles, showing no significant difference. **Right**: The ensemble E3 was found to be more sensitive to predict lesion than SNU-1's model. Overall the models show a fair ability to detect lesions. ^*^p < 0.05, ^**^p < 0.01, and ^***^p < 0.001.

Figure 7 shows that Dice scores of both ensembles were very similarly distributed as the single models' Dice scores. Ensemble E3 did not result in an improved performance, although the median Dice score (0.28) was higher in comparison to ensemble E5 (0.25) and to the winning team SNU-2 (0.26). Likewise, its mean precision was higher (0.34), although not statistically significant, than most single models (SNU-1, SNU-2, UL, INESC). However, the mean sensitivity of E3 (0.51) could be raised over the one from SNU-1 (0.44).

In contrast, ensemble E5 yielded a significantly better mean Dice score (0.31) than UL (0.28, *p* < 0.05) and SNU-1 (0.26, *p* < 0.01). Among the five teams, whose models were used to build the ensemble, SNU-1 was ranked the lowest, explaining why E5 performed significantly better that SNU-1 by itself. While the ensemble's sensitivity was not improved, combining all softmax maps together significantly increased the precision over four single models (*p* < 0.01, INES, SNU-1, SNU-2, UL).

**Figure 8:**
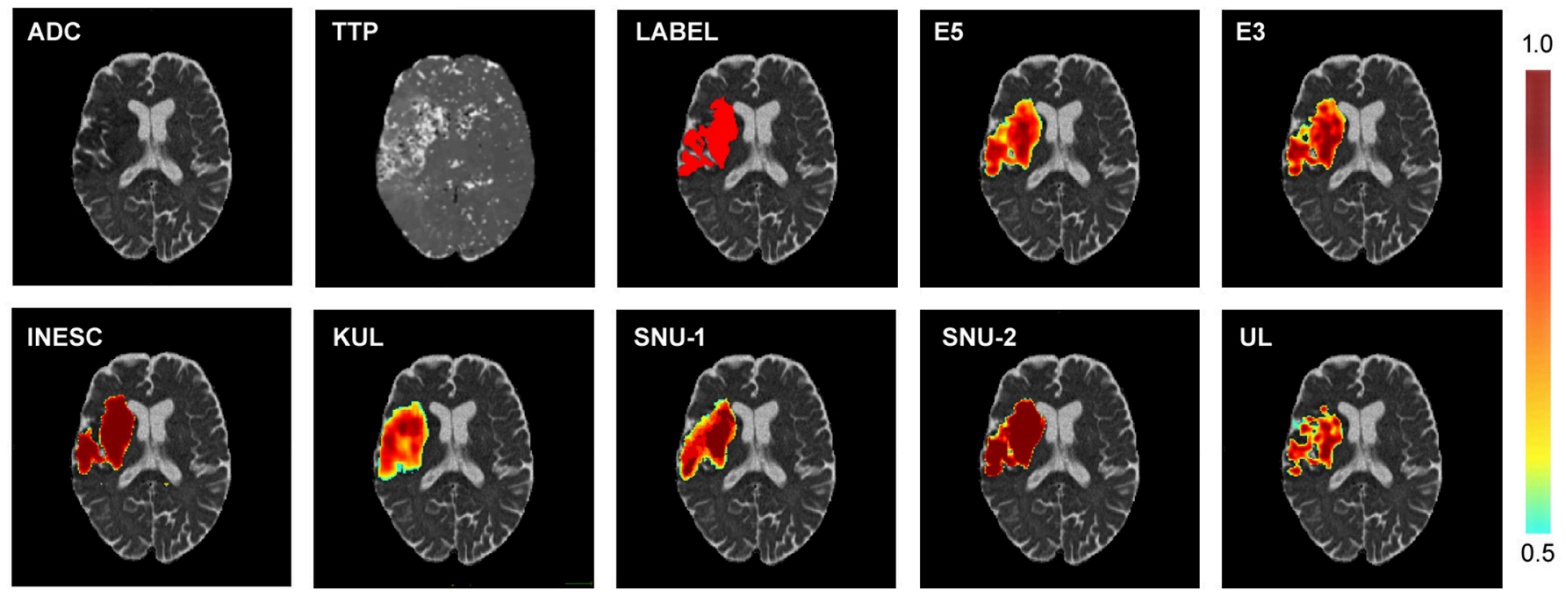
Example of different softmax maps of one patient. Top row: Diffusion (ADC) and perfusion (TTP) scan and the corresponding manual lesion annotation (LABEL) and the softmax maps of the ensembles of the top five (E5) and top three (E3) ranked teams. Bottom row: Softmax maps of the top five ranking teams. Both shape and certainty (see color bar) of the predicted lesion vary between the different participants.

Figure 8 displays an example of the different participants' softmax maps as well as the fused softmax maps of both ensembles (E3 & E5). While softmax maps from INESC and SNU-2 showed similar certainty values through out the predicted lesion, the other three teams' softmax maps appeared to be more heterogeneous. In contrast to the smooth an blob-like structures predicted by SNU-1, SNU-2, INESC and KUL, UL's model provided a greater detail for boundaries. This is also cohesive with the findings, that UL has the highest HD rank (see Table 6) as this metric is considering closeness of boundaries. Dice scores of the lesion predictions for this particular patient could not be improved by ensembles (Dice_*E*5_ = 0.76, Dice_*E*3_ = 0.73) in comparison to the single teams (Dice_*SNU*−1_ = 0.76, Dice_*SNU*−2_ = 0.74, Dice_*UL*_ = 0.60, Dice_*INESC*_ = 0.70, Dice_*KUL*_ = 0.69).

#### 2.2.5. Analysis of robustness of lesiron outcome prediction

We computed Dice scores between the manually outlined lesion groundtruth and differently thresholded and binarised softmax maps for the top five ranking teams. For four teams (SNU-2, UL, INESC & SNU-1) the Dice scores seemed to be fairly robust and centered around the initial threshold of 0.5. SNU-2's and INESC's prediction vary only in about 1 percentage point for different threshold values (see Appendix: Table A1). As an exception, KUL's softmax layer thresholded at a lower level of 0.3 resulted in a higher Dice score (0.28) compared to the the lower Dice (0.26) at a threshold level of 0.5. This effect is coherent with previous findings (see Table 7 and Figure 4) that KUL's produces highly precise predictions with relative low sensitivity. Thresholding at a lower level could assign more voxels to the lesion class, hence increased the model's sensitivity and effectively improve Dice scores.

**Table A1:** Dice score dependency of threshold for softmax maps.

	**Thresholds**								
**Team**	**0.1**	**0.2**	**0.3**	**0.4**	**0.5**	**0.6**	**0.7**	**0.8**	**0.9**
INESC	0.28	**0.29**	**0.29**	**0.29**	**0.29**	**0.29**	**0.29**	**0.29**	**0.29**
KUL	0.22	0.26	**0.28**	0.27	0.26	0.23	0.20	0.15	0.02
SNU-1	0.20	0.23	0.25	0.26	0.26	**0.27**	0.23	0.20	0.16
SNU-2	0.29	0.29	**0.30**	**0.30**	**0.30**	**0.30**	**0.30**	**0.30**	**0.30**
UL	0.19	0.24	0.26	0.27	**0.28**	**0.28**	0.27	0.25	0.21

**Table A2:** Overview of methods of participants of ISLES 2016.

CH-UBE	Random Forest classifier integrating time to reperfusion
DE-UZL	Random Forests classifier
HK-CUH	U-Net architecture; summation instead of concatenation of different pathways
KR-SUC	Ensemble of U-Net architecture and fully convolutional neural network
KR-SUK	
KR-SUL	
PK-PNS	Random Forest classifier
UK-CVI	Combination of CNN and hand-crafted features
US-SFT	U-Net architecture

**Table A3:** Overview of methods of participants of ISLES 2017.

AAMC	3D CNN U-Net architecture; increased number of layers and convolutional filter, multiple down-sampling path ways; anisotropic patch size of 16 × 16 × 4; prediction of 16 overlapping patches per voxels, that are averaged. Morphological operations to reduce small clusters of erroneous predictions
HKU-1	U-Net architecture, including data augmentation and batch normalization, adversarial training of two deep neural networks to avoid over-fitting
HKU-2	3D CNN U-Net architecture; long short-term memory (LSTM) to capture information in 3rd dimension of MRI scans; data augmentation
INESC	V-Net architecture; new loss-function: sum of standard cross-entropy loss and dice-loss
KU	Hierarchy of 2 CNNs. 1st CNN discriminates lesion and healthy tissue, 2nd CNN only acts up on voxels where the 1st CNN was uncertain; auto-context (use of probability maps from 1st CNN)
KUL	U-net architecture; data augmentation via x-axis flip, Gaussian noise and small linear intensity transformations; ensemble of 4 networks; suppression of prediction in non-dominant hemisphere
MIPT	Ensemble of E-Net, DeepMedic, and two U-Nets; 2D and 3D architectures; weighted sum of models' predictions; data augmentation: rotation, flips, registration, and elastic co-registration to template
NEU	Combination 3D U-Net and densely connected CNN; refinement with CRF
NUS	PixelNet applied to lesion outcome prediction
SNU-1/SNU-2	Ensemble of three CNNs: U-Net, DeepMedic, pyramid scene parsing network; negative Dice score loss
SU	3D CNN with 2 scale pathways; data augmentation through rigid transformations, weighted ratios on positive and negative labels
UA	CNN with 4 scale pathways
UL	2D U-Net with skip connections; Dice loss is added up to total loss; inversely weighted loss to tackle class imbalance
UM	2D U-Net in combination with clinical information

#### 2.2.6. Correlation of lesion volumes

When comparing predicted lesion volumes with the manually outlined lesion volumes for the top five ranked teams as mentioned in section 2.2.4, we found a significant correlation only for SNU-1 (Spearman coefficient *r* = 0.39) and for SNU-2 (Spearman coefficient *r* = 0.37). All other teams submission and the ensembles did not correlate with the human rater's annotations, with Spearman coefficients ranging from 0.28 (UL) to 0.35 (E5). As expected, the Dice scores of all models correlated significantly with the lesion volumes, such that the higher the volume the higher the Dice scores. Spearman coefficients were highest for UL (0.72), INESC (0.71) and E3 (0.70), and lowest for KUL (0.41) and SNU-1 (0.55). Mid-range Spearman coefficients were found for SNU-2 (0.59) and E5 (0.68).

## 3. Discussion

### 3.1. Current performance of stroke lesion outcome prediction methods

In ISLES 2016, results showed that deep learning models outperformed *Random Classification Forests* (RF). However, no conclusive superiority of deep learning was found against other machine learning approaches, as demonstrated by CNN-based approaches also ranking in the low tier ranks. Analysing precision and sensitivity revealed the tendency of models to yield over-estimated lesion segmentations. The large variability within the assessed metrics could be explained by the strong correlation between performance and lesion sizes.

Discussions during the ISLES 2016 session led to the decision to enrich the existing ISLES dataset to further encourage participation of the computer science community. Especially, data driven approaches such as deep learning algorithms could truly benefit from larger data sets. Consequently, in ISLES 2017 the training and testing dataset were extended versions of the training and testing sets used in ISLES 2016. For both years, data were provided in minimally pre-processed format. This should should allow a more direct comparison of different stroke prediction models, without the influence of any specific pre-processing steps. Of course advanced processing could foster the tissue outcome prediction, however we argue that our focus for the challenges lies on the model development. Furthermore, the applied pre-processing steps were kept to a minimum and are commonly accepted techniques, such as co-registration. This did not prevent participants to further process the provided data. Although teams also had partly access to raw data (i.e., raw perfusion data), all of them preferred to work with the pre-processed data.

All participating teams of ISLES 2017 suggested a deep learning approach, with top ranked methods featuring CNN architectures. Despite the increased size of the training data, the overall performance was surprisingly not much different than for ISLES 2016. Top ranked models were found to operate on a similar level, sharing similar architectures and system characteristics. Even ensembles of different CNNs were not strong enough to boost the performance further. These results suggest that CNNs' performance may have reached a plateau on this dataset. Future investigation need to strongly focus on improved training strategies for CNNs or on development of new methodologies to advance stroke lesion outcome prediction. Enhancing the performance especially for small sized lesions and incorporating non-imaging information could bear a strong potential for improvement.

It has been shown that ensemble approaches or fusion of results can improve segmentation predictions (14, 15). Our findings suggest that the ensemble approaches had a tendency to perform better than single models. Despite the unimproved sensitivity of the ensembles, combining all softmax maps together significantly increased the precision over four single models. This suggests a reduction of false positive predictions. However the effect was not strong enough to result in statistical improvement over the highest ranked single method. It was also not entirely clear which model contributed to enhance or worsen the performance. In fact, the submissions for ISLES 2017 included single as well as ensembles of neural networks, but the ranking did not reflect an overall superiority of ensemble methods. Although the combination of several weak classifiers can cancel out individual model's limitations, it is nonetheless important to build an ensemble of strong methods to leverage benefits and justify increased computational costs of an ensemble based approaches.

Examining each participating team's softmax maps was motivated to analyse their potential to describe their correctness and certainty to perform the task. As these models are intended to provide a prediction of stroke lesion outcome, we postulate that model calibration is an important aspect for future analysis of deep learning models used for stroke lesion prediction. Particularly, it will have to be investigated how model capacity, regularization and normalization can affect model's calibration, despite apparent increases in model's accuracy (16).

Our findings support the use of different ranking metrics and align with the findings reported in Maier et al. (2). For example the team UL was ranked second in ISLES 2017 thanks to its top HD rank, despite being assigned a relative low Dice rank of 6.16, which would equate the fourth place on the leaderboard.

Overall, the difficulty of the task is reflected by the low Dice scores, with top methods averaging a Dice rank of 0.3. The low Dice scores of the models can be explained by the inherent challenges of the prediction task. Contrary to stroke lesion segmentation, stroke lesion outcome models are trained to predict the lesion status at a 90-days follow-up image based on the acute imaging information. Inherently, many factors contribute to tissue recovery or infarction, which are not explicitly nor implicitly characterized in the imaging information acquired at time of the stroke infarct.

### 3.2. Limitations and remaining challenges

Looking at the evolution of ISLES over the past 3 years, a clear convergence of methodology is observable. While for ISLES 2015 and 2016, still classic machine learning models, such as RF were explored, all submissions of ISLES 2017 offered a variation of CNNs. With their undeniable benefits and success, deep learning methods have set new state-of-the-art benchmarks in many disciplines. Although at present time, this would be the sensible direction to develop further techniques for stroke lesion segmentation and outcome prediction, future challenges will need to encourage exploration of more diverse models. Particularly, we remark the importance of designing methodologies capable of incorporating clinical and physiological prior information on stroke infarction and recovery.

The comparison of the automatic lesion outcome prediction with both expert annotations separately (ISLES 2016) showed a systematic bias toward a higher accordance with rater 2. While this emphasizes the importance of a common database to compare algorithms, it also unveils the general underlying dilemma of supervised learning methods and the intrinsic inter-rater variability observed in medical imaging applications. In best case, algorithms that learn solely from human annotation will only ever be as good as the best human rater and inevitable learn humans' fallacy. Overcoming this limitation calls for semi- and unsupervised learning techniques to teach the computer to detect abnormal brain tissue more accurately, as well as to consider inter-rater variability as source of information during the learning process (17). Nonetheless, a fair and consistent evaluation of such methods has yet to be established. Furthermore, our evaluation is challenged by the different levels of expertise in each team. Although there is a clear tendency that CNNs provide overall better results than RF, some CNNs were ranked lowest. This rather suggest potential deficiencies in the training scheme than a deficiency of this model class in general.

Another challenge is the interpretability of the output of the applied models. Although models are desired to predict lesions with high precision and confidence level, there may lay valuable information in a models uncertainty for clinical decision making. Regarding lesion outcome prediction, uncertainty could give for example a better indicator of tissue at risk of infarction (e.g., naively thought: high certainty means high risk of becoming lesion tissue, while low certainty may reflect tissue likely to be healthy in future). For future challenges we recommend to ask teams to submit non-binary output maps (e.g., softmax maps) that support such analysis. Most methods work indeed best when incorporating multi-parametric information, however, the database will need to be explored, as in Pereira et al. (18) to gain knowledge on which MR sequences are important and to what extent.

## 4. Conclusion

Over the past years, the ISLES team was able to build an increasingly larger MRI database for ischemic stroke lesion MRI. With this publicly available dataset and a continuously open evaluation system, ISLES has the potential to serve as a standard benchmark framework, where researchers can test their algorithms against an existing pool of described and compared methods (14 ISLES 2015 methods for lesion segmentation, and 28 ISLES 2015 & 2016 and 2017 methods for lesion outcome prediction). Despite the great efforts and accomplishments present at ISLES, automatic segmentation of stroke lesions, and more so lesion outcome prediction remain challenging tasks. Deep learning approaches have great potential to leverage clinical routine for stroke lesion patients, but last years of progress at ISLES indicate that further developments are needed to support clinical decision making by incorporating imaging and readily-available non-imaging clinical information, collateral flow modeling, and further improve the interpretability of deep learning systems used for the clinical decision making process of stroke patients.

## Ethics statement

All datasets were fully anonymised through skull-stripping and removal of all patient informations by means of conversion of dice files to nifty volumetric files following the regulations of the Swiss Law for Human Research. Further information added below for sake of completeness (In German). Anonymisierung: Unter anonymisiertem biologischem Material und anonymisierten gesundheitsbezogenen Daten ist die irreversible Aufhebung des Personenbezuges zu verstehen. Eine solche liegt dann vor, wenn Material bzw. Datenüberhaupt nicht oder nur mit einem un-verhältnismässig grossen Aufwand an Zeit, Kosten und Arbeitskraft der betreffenden Person zugeordnet werden können (vgl. Art. 3 Bst. i HFG und Art. 25 Abs. 1 HFV). Wann den Anforde-rungen an eine korrekte Anonymisierung Genüge getan ist, ist je nach Einzelfall zu entschei-den: Die Streichung nur des Namens kann bei einer sehr grossen Datenmenge (grosse Perso-nenpopulation) genügen, auch wenn andere Parameter (z.B. Geburtsjahr) verbleiben. Ist die betroffene Population jedoch sehr klein, so ist das Entfernen nur des Namens nicht ausreichend (vgl. Botschaft zum HFG, S. 8096). Insbesondere unkenntlich gemacht oder gelöscht wer-den müssen Namen, Adresse, Geburtsdatum und eindeutig kennzeichnende Identifikati-onsnummern (Art. 25 Abs. 2 HFV). Das im ursprünglichen Art. 14 HFG (vgl. Botschaft zum HFG, S. 8105) vorgesehene Ver-bot der Anonymisierung von biologischem Material bzw. Personendaten bei Forschungsprojek-ten mit Bezug zu schweren Krankheiten wurde auf Antrag der vorberatenden Kommission vom Nationalrat gestrichen (vgl. Amtliches Bulletin des Nationalrats, 09.079, Verhandlung vom 10.03.2011). Hintergrund war vermutungsweise das in Art. 32 Abs. 3 HFG festgelegte Informa-tions- und Widerspruchsrecht der Patienten bei Forschung mit anonymisiertem biologischen Material und genetischen Daten. Dadurch sind die Patienten nämlich ausreichend geschützt, ein zusästzliches Verbot schien vor diesem Hintergrund wohl obsolet. Mit Streichung des ur-sprünglichen Artikels 14 HFG ist die Forschung mit anonymisiertem biologischem Material also auch bei Forschungsprojekten mit Bezug zu schweren Krankheiten zulässig, sofern die betroffenen Personen vorgängig korrekt informiert und auf ihr Widerspruchsrecht hingewiesen wurden.

## Author contributions

SW, AH, and MR collected data, performed analysis, wrote manuscript. RM, RW, JP, VA, CS, MP, MB, EK, MM, AO, YC, YK, MP, BK, J-HW, MI, HR, DR, PS, YN, EG, JX, JMP, GZ, EK, CL, MH, LC, PA, AB, KC, JB, JK-C, LR, LD and OM performed analysis, wrote manuscript.

### Conflict of interest statement

The authors declare that the research was conducted in the absence of any commercial or financial relationships that could be construed as a potential conflict of interest.

## Footnotes

^1^http://www.isles-challenge.org/ISLES2016/

^2^No softmax maps from team HKU-1 were made available, which is why we include the next ranked team on the list i.e., SNU-1 for E5 and INESC for E3.

## A. Appendix

This following sections briefly summarizes the participants' algorithms.

### A.1. ISLES 2016

#### A.1.1. ISLES16-A1. CH-UBE - incorporating time to reperfusion into the FASTER model of stroke tissue-at-risk

Authors: *Richard McKinley, Roland Wiest, and Mauricio Reyes*

In a recent paper, we introduced the tool FASTER (Fully Automated Stroke Tissue Estimation using Random Forests) (3), which aims to give an assessment of the tissue at risk in acute stroke beyond the usual paradigm of predefined thresholds on single maps. The FASTER system assesses the likelihood of tissue damage using decision forest classifiers, mapping local statistical features of perfusion and diffusion imaging onto maps of the tissue predicted to be lost even if reperfusion is established, and the tissue predicted to be lost only if there is no reperfusion. These models are trained only on extreme cases, in which reperfusion was total and rapid (TICI 3), or completely absent (TICI 0). In this work we attempt to go further, predicting the likely tissue loss in the case of TICI grades 1-2b, by interpolating between the two predictions yielded by FASTER, and incorporating the time to revascularization.

##### A.1.1.1. Acknowledgments

The authors acknowledge the support of the Schweizerische Herzstiftung.

#### A.1.2. ISLES16-A2. DE-UZL - random forests for stroke lesion and clinical outcome prediction

Authors: *Oskar Maier and Heinz Handels*

Ischemic stroke is caused by an obstruction in the cerebral blood supply and, if diagnosed early, part of the under-perfused tissue can potentially be salvaged. Since the available treatment options are not risk-free, the decision has to be made individually, depending on the potential gain and under great time restriction. The prediction of the final lesion outcome in form of A binary mask (Task I) and the prediction of the clinical outcome in form of the modified Rankin Scale (mRS) (Task II) are therefore of great clinical interest. The ISLES 2016 challenge offers a public dataset and associated expert groundtruth to allow researchers to compare their methods in these two fields directly and fairly. Our contribution works with carefully selected features extracted from the MR sequences and used to train a RF. The data consists of multi-spectral (ADC, PWI maps and raw PWI 4D volumes) scans and associated clinical measures. The final lesion outcome as delineated in a 90 days follow-up scan (Task I) and the 90 days mRS score (Task II) serve as groundtruths. More details on the data can be found on www.isles-challenge.org. Task I: Lesion outcome prediction From each MR sequence we extract the features previously presented in (32), but furthermore employ a hemispheric difference measure to make use of the pseudo-quantitative values provided by the PWI maps. For voxel-wise classification we employ RFs. Task II: Clinical outcome prediction Based on the segmentation results from Task I, we extract lesion characteristics as well as local image features from the supplied cases to train a regression forest. Applied, this yields a prediction of the mRS score for a formerly unseen case. Our method has been shown to provide competitive lesion segmentation results in glimo segmentation as well as acute and semi-acute stroke in the previous year's edition of the ISLES challenge. The results from this year's challenge will show if the advantages of our flexible design also extend to outcome prediction.

#### A.1.3. ISLES16-A3. HK-CUH - residual volumetric network for ischemic stroke lesion segmentation

Authors: *Lequan Yu and Pheng-Ann Heng*

We propose a 3D CNNs based method for lesion outcome prediction. The proposed 3D network takes advantage of fully convolutional architecture to perform efficient, end-to-end, volume-to-volume training. More importantly, we introduce the recent proposed residual learning technique into our network, which can alleviate vanishing gradients problem and improve the performance of our network. It employs 3D fully convolutional architecture and is organized in a residual learning scheme. The layers of our network are all implemented with a 3D manner (under caffe library), thus the network can highly preserve and deeply exploit the 3D spatial information of the input volumetric data. We adopt small convolution kernels with size of 3 × 3 × 3 in convolutional layers. Each convolutional layer is followed by a rectified linear unit (ReLU). Note that we also employ batch normalization layer (BN) before each ReLU layer. The BN layer can accelerate the training process of our network. At the end of the network, we add a 1 × 1 × 1 convolutional layer as a classifier to generate the segmentation results and further get the segmentation probability volumes after passing the softmax layer. Note that our network might appear similar to U-Net, but there are differences: We use summation units instead of concatenation units when combining different paths, and thus we can reformulate our network as residual learning scheme; additionally, we adopt recently developed batch normalization technique to improve our performance.

#### A.1.4. ISLES16-A4. KR-SUC/KR-SUK/KR-SUL - deep convolutional neural network approach for brain lesion segmentation

Authors: *Youngwon Choi, Yongchan Kwon, Hanbyul Lee, Myunghee Cho Paik, and Joong-Ho Won*

Brain lesion segmentation is a challenging problem because the amount of lesion area is extremely small and the size of available training magnetic resonance images are limited. To handle this, we exploit millions of 3D patches and 3D convolutional kernels for our proposed model. By treating each 3D patch as training data we capitalize on spatial information and overcome the problem of limited medical data. Our final segmentation model is an ensemble of two deep convolutional neural networks inspired by fully convolutional networks and the U-Net (36). We implement the proposed model in Python with Lasagne and Keras.

#### A.1.5. ISLES16-A5. PK-PNS - segmentation of ischemic stroke lesion using random forests in multi-modal MRI images

Authors: *Qaiser Mahmood and A. Basit*

Multi-modal MRI can be used for detecting the ischemic stroke lesion and can provide quantitative assessment of lesion area. It can be established as an essential paraclinical tool for diagnosing stroke. For a quantitative analysis of stroke lesion in MRI images, clinical expert manual segmentation is still a common approach and has been employed to compute the size, shape, and volume of the stroke lesions. However, it is time-consuming, tedious, and labor-intensive task. Moreover, manual segmentation is prone to intra-and inter-observer variabilities. Herein, we present an automated segmentation method for ischemic stroke lesion segmentation in multi-modal MRI images. The method is based on an RF ensemble learning technique called random forest, which generates several classifiers and combines their results in order to make decisions. In RF, we employ several meaningful features such as intensities, entropy, gradient etc. to classify the voxels in multi-modal MRI images. The segmentation method is validated on training data, obtained from MICCAI ISLES-2016 challenge dataset. The performance of the method is evaluated relative to the manual segmentation, done by the clinical experts. The experimental results show the robustness of the segmentation method, and that it achieves reasonable segmentation accuracy for segmenting the ischemic stroke lesion in multi-modal MRI images.

#### A.1.6. ISLES16-A6. UK-CVI - combination of CNN and hand-crafted feature for ischemic stroke lesion segmentation

Authors: *Haocheng Shen, Siyamalan Manivannan, Roberto Annunziata, Ruixuan Wang and Jianguo Zhang*

Convolutional neural networks can automatically learn discriminative local features and give superior performance than hand-crafted features in various applications such as image classi-fication, semantic segmentation and object detection. CNN has also been applied to MRI brain image analysis and achieved state-of-the-art results for brain tumor region segmentation (22, 7), stroke lesion segmentation (7), and mircobleeds detection (28). Recently, some studies [e.g., (23)] show that hand-crafted features may provide complementary information with CNN, hence combining them with the features extracted from CNN may give improved performance than only using the features from CNN. Motived by this, we formulate the segmentation of ischemic stroke lesion in acute MRI scans as a pixel-level classification using a combination of CNN and hand-crafted features. We used a CNN architecture which is similar to (38). It is a fully convolutional neural network containing a downsampling path and three upsampling paths. In the task of stroke lesion segmentation, there is a large variation on the size, location, and shape of lesions. Therefore, encoding information at multiple scales is necessary and preferable than considering information at only one level. The downsampling path is able to extract the abstract information with high-level semantic meaning, while the three upsampling paths are designed to capture the fine details. These three upsampled feature maps are then combined at the later stages of the CNN architecture so that the classification layer fully make use of the information appears at multiple scales (38). We use the following hand-crafted features: intensity, the hemispheric intensity difference between two symmetric pixels in the axial view, first order statistics in a w × w volume patch, maximum response filter (MR8) (34). At each 2D pixel location, these local features are extracted independently from each image modality and combined together to get a feature representation for that pixel. As there is a large variation of lesions in the dataset, it will be beneficial to train a pool of binary classifiers instead of one. Each binary classifier in this pool is designed to separate the positive (lesion) features extracted from a patient from all the negative (normal) features extracted from the same patient. In this way we believe that some rarely appeared lesions can be easily discriminated from the normal tissue compared to a binary lesion classifier which is trained using all the training data (without using patient information). In the testing time a voting strategy (averaging the top 3 probabilities obtained by the binary classifiers in the pool) is used to get the prediction of an input.

#### A.1.7. ISLES16-A7. US-SFT - a deep-learning based approach for ischemic stroke lesion outcome prediction

Authors: *Ramandeep Randhawa, Ankit Modi, Parag Jain, and Prashant Warier*

The ISLES 2016 challenge aims to address two important aspects of Ischemic stroke lesion treatment prediction. The first aspect relates to segmenting the brain MRI to identify the areas with lesions and the second aspect relates to predicting the actual clinical outcome in terms of the patient's degree of disability. The input data consists of acute MRI scans and additional clinical such as TICI scores, Time Since Stroke, and Time to Treatment. To address this challenge we take a deep-learning based approach. In particular, we first focus on the segmentation task and use an automatic segmentation model that consists of a Deep Neural Network (DNN). The DNN takes as input the MRI images and outputs the segmented image, automatically learning the latent underlying features during the training process. The DNN architectures we consider utilize many convolutional layers with small kernels, e.g., 3 × 3. This approach requires fewer parameters to estimate, and allows one to learn and generalize from the somewhat limited amount of data that is provided. One of the architectures we are currently utilizing is based on the U-Net (36), which is an all-convolutional network. It acts as an auto-encoder, that first “encodes” the input image by applying combinations of convolutional and pooling operations. This is followed by the “decoding” step that up-scales the encoded images, while performing convolutions. The all-convolutional architecture of the U-Net allows it to handle input images of different dimensions as in the challenge dataset. In our experiments, we found that this architecture yielded excellent performance on the previous ISLES 2015 dataset. Although the modalities in the 2016 challenge are different, our initial training experiments have yielded promising segmentation results. Our next steps involve addressing the regression challenge. There is limited amount of labeled data for this task. Our approach will be to include these outcomes as part of the segmentation training directly. This will allow the DNN to learn latent features that can directly help with the classification task.

### A.2. ISLES 2017

#### A.2.1. ISLES17-A1. AAMC - ensembling 3D U-nets for ischemic stroke lesion segmentation

Authors: *Andrew Beers, Ken Chang, James Brown, Emmett Sartor, Elizabeth Gerstner, Bruce Rosen, and Jayashree Kalpathy-Cramer*

We propose a novel deep learning architecture based on the 3D Convolutional U-Net, an architecture that has found success both in ISLES 2016 and a wide array of other tissue segmentation applications. A typical U-Net segmentation architecture operates by convolving and downsampling input data stepwise into a low-resolution representation, and then upsampling and deconvolving that representation into to a categorical labelmap. The downsampling arm of the U-Net is also concatenated at points to the upsampling arm, resulting in a densely-connected architecture. We improve upon previous implementations of the 3D U-Net both by increasing the number of layers and convolutional filters, and by adding multiple independent down-sampling arms to the network. The motivation for this chimeric structure is to increase accuracy by concatenating several unique and not necessarily correlated downsampled representations, thereby increasing the potential amount of relevant imaging biomarkers. We apply this architecture on stacked, 16 × 16 × 4 voxel patches of six of the seven given image maps (ADC, CBV, CBF, MTT, TTP, Tmax) for ISLES 2017. For training, 80% of patches are drawn from the groundtruth regions, while 20% of patches are extracted from normal brain. For inference, we predict 16 overlapping output patches per voxel, average overlapping softmax outputs, and threshold those outputs into binary labels. We finally post-process the binary labels by removing small islands and applying repeated segmentation erosions and dilations.

##### A.2.1.1. Acknowledgments

We would like the acknowledge the GPU computing resources provided by the MGH and BWH Center for Clinical Data Science.

#### A.2.2. ISLES17-A2. HKU-1 - deep adversarial networks for stroke lesion segmentation

Tony C. W. Mok and Albert C. S. Chung

Training models that provide accurate stroke lesion segmentation for stroke assessment is challenging. Methods based on deep convolutional neural networks usually rely on large amounts of annotated data. The small lesion area and limited size of available acute MRI data would degrade the quality of result using such approaches due to over-fitting the training data. To deal with this problem, we adopt two deep neural networks with adversarial training (25). (31) shows that this technique could generate a regularization effect and result in less over-fitting to the training data. Our model ensemble two deep convolutional neural networks inspired by the U-Net (36). Other technique such as data augmentation and batch normalization are adopted to further improve the final results.

#### A.2.3. ISLES17-A3. HKU-2 - stochastic dense network for brian lesion segmentation

Authors: *Pei Wang and Albert C. S. Chung*

The segmentation of ischemic stroke lesion in brain MRI is quite challenging for its varying size and unknown shape. To tackle this problem, we proposed a convolutional neural network for an end-to-end, volume-to-volume lesion segmentation. Based on the 3D U-Net structure, we apply dense connection to link every two layers to well combine the low level information with the high level one. In each layer, instead of 3D convolution, we adopt long short-term memory (LSTM) to capture the information of third dimension in MRI. To further reduce the over-fitting during training process, all the dense connections between layers are stochastically established. Due to the limited dataset, data augmentation is applied to the training dataset.

#### A.2.4. ISLES17-A4. INESC - fully convolutional neural network for 3D stroke lesion segmentation

Authors: *Miguel Monteiro1 and Arlindo L. Oliveira*

Our approach consists of a Fully-Convolutional Neural Network (FCNN) with a V-Net (33) architecture. The V-Net architecture is a variation of the U-Net architecture (36) which is commonly used for medical imaging segmentation. This architecture consists of a contracting path and an expanding path each made up of convolution blocks. At each level of the contracting path, the image's spatial dimensions are halved and the number of channels is doubled. In the expanding path, the opposite happens. There are skip-connections between the contracting and expanding path which feed high-resolution features to the expanding path. In addition, the convolution blocks in both paths have skip connections similar to those of the ResNet (39) which make training faster and more robust. To address class imbalanced (most of the voxels are labeled as 0 in the segmentation) we proposed a novel loss function to train the network. This loss function consisted of the sum of the standard cross-entropy loss with the dice-loss. The dice-loss is calculated by taking the negative dice coefficient calculated with label probabilities instead of discrete labels which results in a number between –1 and 0. Since the cross-entropy loss can take any positive value up to infinity, during training, it begins by dominating the overall loss function. As training progresses, it tends toward 0, at this point the dice-loss component becomes more dominant which helps fine tune the prediction.

##### A.2.4.1. Acknowledgments

This work was supported by PAC - PRECISE - LISBOA-01-0145-FEDER-016394, co-funded by FEDER through POR Lisboa 2020 - Programa Operacional Regional de Lisboa PORTUGAL 2020 and Fundação para a Ciência e a Tecnologia.

#### A.2.5. ISLES17-A5. KU - gated two-stage convolutional neural networks for ischemic stroke lesion segmentation

Authors: *Jee-Seok Yoon, Eun-Song Kang, and Heung-Il Suk*

We propose a novel framework with a gated two-stage CNN for ischemic stroke lesion segmentation. Specifically, there are two CNNs in our framework. The first CNN produces a probability of being normal tissue, i.e., normal, or being ischemic stroke lesion, i.e., lesion. Based on our observation that as for the misclassified voxels in images, the ratio between probabilities of normal and lesion was low. That is, when the probabilities of normal and lesion are close to each other, it can be a good indicator of low confidence to make a decision. In this regard, we devise a gate function that computes the probability ratio between normal and lesion. When the ratio is lower than a threshold, the gate function turns on the second CNN to operate. It is noteworthy that in our second CNN, we also utilize the probabilities obtained from the first CNN as context information. In our experiments, we could validate the effectiveness of the proposed two-stage CNN architecture.

##### A.2.5.1. Acknowledgments

This work was supported by Institute for Information & Communications Technology Promotion (IITP) grant funded by the Korea government (No. 2017-0-00451).

#### A.2.6. ISLES17-A6. KUL - dual-scale fully convolutional neural network for final infarct prediction

Authors: *David Robben and Paul Suetens*

We perform a voxelwise classification to predict the final infarct using relative time-to-peak, ADC and the available metadata. Relative time-to-peak is calculated per voxel as the time-to-peak (TTP) minus the first quartile of the TTP within the brain mask. The given modalities have physical units that can be interpreted absolutely, hence we use per modality the same linear transformation for all subjects: subtraction by the median mean value and scaling with the median standard deviation. The metadata are normalized similarly, after converting the TICI score into a numerical value. Inspired by (7) we implement using Keras a fully convolutional neural network with two pathways, one on the original resolution and one on a lower resolution (in plane subsampled with a factor 3). Both pathways have five 3 × 3 × 1 kernels and five 3 × 3 × 3 kernels to account for the anisotropy of the voxel size. Both pathways and the metadata are subsequently fed into two fully connected layers before the final classification is made. The network is regularized with drop-out and l2-regularization. We augment the training data with flips along the x-axis, Gaussian noise and small linear intensity transformations. Hyperparameters are chosen by evaluating the network's performance during cross-validation on the training set. Training is stochastic and at testing time we use an ensemble of four networks whose predictions are averaged. The predictions are thresholded at 0.5 and all voxels on the non-dominant side of the brain are suppressed.

#### A.2.7. ISLES17-A7. MIPT - neural networks ensembles for ischemic stroke lesion segmentation

Authors: *Maxim Pisov, Mikhail Belyaev, and Egor Krivov*

We use four different architectures of CNNs for image segmentation: a modification of ENet (20), DeepMedic (7), and two versions of U-Net (36). ISLES-2017 problem is a challenging task because of a strong anisotropy of the data: a typical voxel size is about 1 × 1 × 6*mm*^3^. That's why we used E-Net and U-Net as 2D-segmentation networks: 2D slices along the axial plane were fed into them at both training and inference steps, while DeepMedic was used as a 3D-segmentation network. Based on these network architectures we built several models with different hyper-parameters. The masks predicted by these models had significantly variable geometrical properties, e.g., smooth/rough edges, smaller/bigger regions. To reduce this variability, we used a weighted sum of final models' predictions. As a preprocessing step, we cropped all the brain images to their bounding boxes and rescaled them to the shape 192 × 192 in the plane *xOy*. To overcome the dataset size limitations, we use two different data augmentation techniques: classical spatial transformations (e.g., random rotations, random flips along the coronal, and sagittal planes) and a new co-registration-based method. The main idea of the method is to map lesions from a brain with stroke to a healthy brain using elastic co-registration. To augment data in that way we used the approximately age-matched brains of healthy subjects from the Alzheimer's Disease National Initiative dataset (adni.loni.usc.edu) as templates and applied the co-registration algorithm from ANTs toolkit (26).

#### A.2.8. ISLES17-A8. NEU - combination of U-Net and densely connected convolutional networks

Authors: *Donghyeon Kim, Joon Ho Lee, Dongjun Jung, Jong-min Yu, and Junkil Been*

Brain lesion segmentation is an advanced challenging problem which has been handled by only experienced clinician and could not be localized using a single brain imaging method. Thus, it is essential to analyze it as multi modality sense. To address this challenge, we take convolutional neural network, specially U-Net (36), 3D U-Net (24), and Densely Connected Convolutional Network (35). In feature selection, first of all, we searched the best combination of multi data sets and the best number of convolutional neural layers considering computation cost, accuracy, and overfitting problem. With different numbers of image dataset combination, each different image of training data is ensembled to learn at the front of the bridge part between encoding (convolution layer) and decoding (deconvolution layer) in the proposed network. Furthermore, we consider the type of data extraction of the images (2D and 3D patch) and refining the result such as conditional random field (CRF).

#### A.2.9. ISLES17-A9. NUS - fully convolutional network with hypercolumn features for brain lesion segmentation

Authors: *Mobarakol Islam and Hongliang Ren*

The segmentation of stroke lesion is very necessary for diagnosis, planning treatment strategies and monitoring disease progression. We propose a fully convolutional network (FCN) with hypercolumns features and sparse pixel predictions (e.g., PixelNet) for automatic brain lesion segmentation. PixelNet extracts feature from multiple layers that correspond to the same pixel and samples a modest number of pixels across a small number of images for each SGD (Stochastic gradient descent) batch update. Deep Learning (DL) models like CNN requires large training data to generalize the model where most of the biomedical problems have small available dataset. Moreover, the problem of label imbalance leads the CNN often converge to the certain labels. PixelNet deals these problems by utilizing sparse pixel prediction on a modest number of pixels. We utilize PixelNet in ISLES (Ischemic Stroke Lesion Segmentation) challenge 2017 and achieve 68% Dice accuracy as preliminary result.

#### A.2.10. ISLES17-A10. SNU-1 & SNU-2 - schemic stroke lesion segmentation with convolutional neural networks for small data

Authors: *Youngwon Choi, Yongchan Kwon, Myunghee Cho Paik, Beom Joon Kim, and Joong-Ho Won*

Our approach to the ISLES 2017 challenge was to build an ensemble of three-dimensional CNN models predicting ultimate ischemic stroke lesions from early imaging. We employed three types of CNNs: (I) multiscale U-net (24), (II) multiscale fully-convolutional network (7, 37), and (III) pyramid scene parsing network (19). Negative Dice score, binary crossentropy and weighted binary cross-entropy (21) were used as the loss for training. The multiscale U-net architecture trained with the negative Dice score achieved the best performance among the nine combinations considered. The implementation details such as pre-processing, data augmentation, and regularization are similar to (30), which ranked the 1st place in ISLES 2016. There are two major improvements from our approach to the 2016 challenge. First, the model complexity is reduced by 60% without sacrificing the prediction performance: multiscale U-net with 40,000 parameters showed comparable performance to the 2016 model with 100,000 parameters. Second, the training process is simplified by adopting probability calibration instead of the fine-tuning step in the multiphase training (22).

##### A.2.10.1. Acknowledgments

This research was supported by the National Research Foundation of Korea (NRF) grant funded by the Korean government (MSIT, NRF-2016R1C1B1012002). Joong-Ho Won's research was partially supported by the National Research Foundation of Korea (NRF) grant funded by the Korean government (MSIT, No. 2014R1A4A1007895). Myunghee Cho Paik's research was supported by the National Research Foundation of Korea under grant NRF-2017R1A2B4008956.

#### A.2.11. ISLES17-A11. SU - multi-scale patch-wise 3D CNN for ischemic stroke lesion segmentation

Authors: *Yilin Niu, Enhao Gong, Junshen Xu1, John Pauly, and Greg Zaharchuk*

A deep network model was trained with 3D CNN patch-wise approaches and multi-scale structures. A three-dimensional CNN was implemented to utilize available spatial information efficiently and exploit the relationship between slices. Our patch-wise approach extracts concentric small 3D patches from multi-contrast input volumes to emphazise local voxel information, minimize unrelated distant features and handle various volume dimensions. Overlapping 3D patches were sampled from brain regions (using brain masks) at multiple scales (with 2 scale pathways using 36x36x5 and 16x16x3 patch size in the final implementation) to capture both local and global contextual information simultaneously (7). Rigid transformations were used for data augmentation and weighting ratios on positive and negative labels were added to ensure better data balance. The model we implemented has 7 layers, including 1 resample layer right after the inputs, 5 convolutional layers without pooling, 1 resample layer to ensure consistent resolution of the outputs from two scale pathways and 2 fully-connected layers to generate final 6 × 6 patch outputs. From the 43 cases in the training dataset, we split labeled data into 77% for training and 23% for validation. The Dice Score Coefficient was used as training loss and quality metrics in validation. The model is trained using tensor-flow framework on a Linux server with 2 NVIDIA GTX-1080TI GPUs.

#### A.2.12. ISLES17-A12. UA - volumetric multimodality neural network for ischemic stroke segmentation

Authors: *Laura Silvana Castillo, Laura Alexandra Daza, Luis Carlos Rivera, and Pablo Arbeláez*

High level research architectures for semantic segmentation, such as VGG (27) and FCN (37), take advantage of multiple image resolutions to simultaneously extract fine details and coarse structures from the input data by using groups of convolutional layers and non-linearities, usually Rectified Linear Units (ReLU), followed by pooling operations. However, as the resolution of the image is reduced, so is the accuracy in the segmentation location. To overcome this drawback, we propose a neural network that extracts features from different input resolutions in a parallel and independent manner. Additionally, the use of a patch-wise approach helps to deal with the imbalance of the data and reduces the memory consumption. This allows us to retrieve detailed appearance data along with accurate semantic information simultaneously. Our method is based on DeepMedic (7) and V-Net (33), methods that have shown state of the art on medical image segmentation. We developed a new architecture with four parallel pathways, each one with six convolutional layers and two residual connections, to extract features on specific resolution levels. All the paths receive patches centered at the same voxel, but extracted from different versions of the image (original and downsampled by factors of six and eight). The patches have input sizes of 363, 203, 183, and 153 for the normal, medium and low resolution pathways. An upsample layer is used to make the outputs of the same size. Finally, the results are concatenated and introduced in fully connected layers to be combined and then classified. The classification layer is a convolution with kernel size of 13.

#### A.2.13. ISLES17-A13. UL - 2D multi-scale res-net for stroke segmentation

Authors: *Christian Lucas and Mattias P. Heinrich*

U-Nets (36) have shown competitive performance in different biomedical tasks while being capable of segmenting objects of different scales. Ischemic strokes vary widely in location, shape, and extend of the affected tissue. We thus propose a fully-convolutional architecture based on U-Nets for segmenting transversal image slices. The challenge data has been resampled to a common resolution of 1 × 1 × 5mm and slices are zero-padded, if required. The network is provided 42 image features as input (7 MR sequences, 3 slices including both direct neighboring slices, 2 hemispheric flips). In the contracting path, fine-grained information is improved across the five scale levels of the U-Net (from 240 × 240 down to 15 × 15) by additional skip connections: the input of each level is concatenated channel-wise with the activation [similar to ResNets (29) but with concatenation] before it is downsampled and passed to the deeper level. In the upscaling path, the Dice loss at each level is computed on softmax activation and summed up to a total loss for training. The loss of foreground and background is weighted with its inverse prior probability (estimated from training data) to account for class imbalance. To speed up training, the network parameters are optimized using the ADAM algorithm. Moreover, each convolution (in both paths) is followed by a batch normalization as done before in Lucas et al. (6).

##### A.2.13.1. Acknowledgments

This work was supported by the Graduate School for Computing in Medicine and Life Sciences funded by Germany's Excellence Initiative [DFG GSC 235/2]. We would also like to thank Nvidia Corporation for their support by providing us with a Titan Xp graphics card.

#### A.2.14. ISLES17-A14. UM - combining clinical information for stroke lesion outcome prediction using deep learning

Authors: *Adriano Pinto, Richard Mckinley, Victor Alves, Roland Wiest, Carlos A. Silva, and Mauricio Reyes*

For stroke lesion outcome prediction, we propose an end-to-end deep learning method capable of merging MRI sequences with non-imaging clinical information, namely the thrombolysis in cerebral infarction (TICI) scale. Since MRI images come from different centers, as preprocessing steps we resized all MRI sequences to 256 × 256 × 32. In addition, the Tmax sequence was clipped to [0, 20*s*] and the ADC sequence was clipped within the range of [0, 2600] × 10^−6^*mm*^2^/*s*, as values beyond these ranges are known to be biologically meaningless (3). Afterwards, all sequences were linearly scale to [0, 255]. Our architecture has two main blocks, the first is based on the 2D-Unet (36), whose output feature maps are injected in a second block composed by two layers of Gated Recurrent Units (41). The clinical domain knowledge is incorporated at two levels: population and patient levels. The population level is coded in a custom loss function based on the *F*_β_−*score* (40), having the beta parameter modeled by the TICI scale. To encompass this clinical knowledge into the testing phase we added an extra input channel that contains the TICI score. Therefore, we aim to drive the learning process of the architecture accordingly to the success of revascularization, in order to produce optimist predictions when the predicted lesion shrinks, and pessimistic predictions when the predicted lesion increases.

##### A.2.14.1. Acknowledgments

Adriano Pinto was supported by a scholarship from the Fundação para a Ciência e Tecnologia (FCT), Portugal (scholarship number PD/BD/113968/2015). This work is supported by FCT with the reference project UID/EEA/04436/2013, by FEDER funds through the COMPETE 2020 - Programa Operacional Competitividade e Internacionalização (POCI) with the reference project POCI-01-0145-FEDER-006941. We acknowledge support from the Swiss National Science Foundation - DACH 320030L 163363.
